# Utility of wide-area transepithelial sampling with 3-dimensional computer-assisted analysis as an adjunct to forceps biopsy sampling in the surveillance of patients with Barrett’s esophagus after endoscopic eradication therapy

**DOI:** 10.1016/j.igie.2022.10.011

**Published:** 2022-11-03

**Authors:** F. Scott Corbett, Robert D. Odze, Matthew J. McKinley

**Affiliations:** 1 Department of Medicine, Florida State University College of Medicine, Sarasota, Florida, USA; 2 Division of Gastroenterology, Sarasota Memorial Hospital, Sarasota, Florida, USA; 3 Suncoast Endoscopy of Sarasota, Florida Digestive Health Specialists, Sarasota, Florida, USA; 4 Department of Pathology, Tufts University School of Medicine, Boston, Massachusetts, USA; 5 Department of Medicine, NYU Grossman School of Medicine, New York, New York, USA

## Abstract

**Background and Aims:**

Recurrence of Barrett’s esophagus (BE) and associated dysplasia is not uncommon after endoscopic eradication therapy (EET). However, the most optimal method for detection of residual/recurrent disease remains unknown. This study evaluated the utility of wide-area transepithelial sampling with 3-dimensional computer-assisted analysis (WATS-3D) as an adjunct to standard forceps biopsy (FB) sampling for detection of BE and dysplasia after EET.

**Methods:**

Data were examined from 2 large commercial registries of community practices that prospectively evaluated BE surveillance using both standard FB sampling combined with WATS-3D. Comprising the study cohort were 1114 consecutive BE patients (mean age, 68.9; men 68.1%, women 31.9%) who had EET. The adjunctive (and absolute) increased yield of WATS-3D for detection of intestinal metaplasia (IM) and dysplasia was evaluated according to the extent of endoscopic residual BE.

**Results:**

The WATS-3D adjunctive yield for detection of residual/recurrent IM or dysplasia was 52.5% and 91.5%, respectively. The highest adjunctive yield for detection of IM (260%) occurred in patients without any gross endoscopic evidence of residual or recurrent BE. The absolute yield for IM and dysplasia detection was 16% and 4.4%, respectively, and dysplasia detection was significantly greater in patients with visible BE compared with those with no endoscopic evidence of residual BE. Of 29 patients with high-grade dysplasia or esophageal adenocarcinoma detected by WATS-3D, FB sampling missed 11, including 7 where FB sampling did not detect any IM. The number of patients needed to test for detection of residual/recurrent BE was 6.2 and for detection of dysplasia was 22.9.

**Conclusions:**

WATS-3D is effective at increasing the diagnostic yield of IM and dysplasia in BE patients after EET when used as an adjunct to FB sampling.

Barrett’s esophagus (BE) is a chronic metaplastic condition because of GERD that develops from conversion of the normal squamous lined epithelium of the esophagus into one that is columnar, often with intestinal metaplasia (IM).^1^^,^^2^ Patients with BE are at a greatly increased risk of developing esophageal adenocarcinoma (EAC) through the metaplasia–dysplasia–cancer sequence.^3^, ^4^, ^5^, ^6^, ^7^, ^8^

Current GI societal guidelines recommend treatment with endoscopic eradication therapy (EET) for BE patients with dysplasia or stage T1a intramucosal adenocarcinoma.^9^^,^^10^ This form of treatment has been shown to be cost-effective with results comparable with esophagectomy for early EAC in terms of outcome.^9^^,^^10^ Radiofrequency ablation is considered the first-line choice of EET by most GI societies worldwide because of its high efficacy and safety record.^10^, ^11^, ^12^, ^13^, ^14^, ^15^, ^16^, ^9^ However, recurrences are not uncommon. For instance, fairly recent studies have demonstrated that recurrent (or residual) IM and dysplasia occur at a rate of approximately 8% to 10% for the former and 2% to 3% for the latter per patient year of surveillance.^9^^,^^17^, ^18^, ^19^, ^20^, ^21^, ^22^

Recurrences occur in the tubular esophagus but are more common at or within 3.0 cm of the esophagogastric junction (EGJ) or cardia. Recurrence is generally classified according to whether it is endoscopically visible (most) or not, the latter only detected by histologic analysis of forceps biopsy (FB) sampling.^9^^,^^18^^,^^19^ However, both residual and recurrent IM and dysplasia can be subtle and difficult to detect endoscopically, especially surrounding the EGJ. Furthermore, the precise method of obtaining biopsy samples is controversial and not yet supported by prospective clinical trials. Regardless of the method used, it is known that compliance with preablation surveillance biopsy sampling protocols, such as the Seattle protocol,^23^ is suboptimal and that even extensive biopsy sampling protocols are known to sample only a small fraction of the mucosa. Thus, sampling error continues to be a clinical problem after EET.^24^, ^25^, ^26^, ^27^

Wide-area transepithelial sampling with 3-dimensional computer-assisted analysis (WATS-3D) is a diagnostic platform that allows for widespread sampling of the esophagus with the use of a unique brush instrument designed to sample deep aspects of the mucosa, combined with 3D imaging and neural network analysis of the smear specimen specifically developed to recognize both IM and dysplasia.^28^, ^29^, ^30^ Multiple prior prospective studies have suggested a significant increase in detection of BE and associated dysplasia when WATS-3D is used as an adjunct to FB sampling in patients undergoing screening or surveillance of BE.^29^, ^30^, ^31^, ^32^, ^33^ Although the efficacy of this technique in postablation surveillance has been addressed in abstract form,^34^ it has not been published in a peer-reviewed study. Thus, the purpose of this study was to evaluate the diagnostic efficacy of WATS-3D for detection of IM and dysplasia/EAC in BE patients who have undergone EET.

## Methods

The study comprised 1114 BE patients, all of whom were either undergoing or had completed EET (Table 1) and received at least 1 postablation surveillance endoscopy with both FB sampling and WATS-3D for detection of residual IM and/or dysplasia (where applicable). In total, 1715 endoscopic procedures were performed.

**Table 1:** Distribution of demographic and clinical characteristics of patients on their first visit (n = 1114)

Characteristics	Value
Age, y	
Mean (standard deviation)	68.9 (10)
Median [minimum, maximum]	70.3 [29.4, 93.1]
Sex	
Male	759 (68.1)
Female	355 (31.9)
Race/ethnicity	
White	980 (88.0)
Hispanic/Latino	34 (3.1)
Black/African American	22 (2.0)
Asian	10 (.9)
Native American /Alaska Native	3 (.3)
Unknown	65 (5.8)
Ablation method	
Radiofrequency ablation	866 (77.7)
Combination ablation treatment∗	119 (10.7)
Cryoablation therapy	28 (2.5)
Argon plasma coagulation	2 (.2)
Unknown	99 (8.9)
Worst preablation pathology	
Intestinal metaplasia	534 (47.9)
Indefinite dysplasia	54 (4.8)
Crypt dysplasia	7 (.6)
Low-grade dysplasia	278 (25.0)
High-grade dysplasia/esophageal adenocarcinoma	208 (18.7)
Dysplasia, not specified	33 (3.0)
Hiatal hernia	
Small	13 (1.2)
Moderate	139 (12.5)
Large	101 (9.1)
Present, unknown size	419 (37.6)
Not present	413 (37.1)
Unknown	29 (2.6)
Postablation salmon-colored mucosa length (1715 endoscopies)	
0 cm	205 (12.0)
.1-.9 cm	687 (40.1)
1-2.9 cm	608 (35.5)
≥3 cm	215 (12.5)

Values are n (%) unless otherwise indicated.

∗ Combination ablation treatments included radiofrequency ablation (RFA)/cryoablation therapy, cryoablation therapy/argon plasma coagulation (APC), RFA/APC, RFA/APC/cryoablation therapy, and RFA/photodynamic therapy/APC.

Patients were derived from a pooled analysis of 2 large prospective commercial-based observational registry studies (nos. 706 and 806; CDx Diagnostics, Suffern, NY, USA) who had received a WATS-3D test from September 20, 2015 to October 20, 2020 from 121 community-based physicians across the United States. Institutional review board approval was obtained from every site (Solutions Institutional Review Board approval 706 7/27/2015 and 806 2/27/2016).

Patients were enrolled in our analysis if they were being surveyed for BE after EET (≥1 EET session) and did not have prior esophageal surgery other than EMR. All patients underwent both WATS-3D and FB sampling at the time of postablation endoscopy. The WATS-3D database from these registries contains clinical, endoscopic, and histologic data regarding all samples sent for WATS-3D analysis. The study cohort included patients whose baseline diagnosis preablation was nondysplastic BE (NDBE), most of whom were derived from 2 large studies specifically looking at this group as well as patients with dysplasia. Additionally, the cohort included patients who had not yet completed EET as well as some deemed refractory to EET. Patients were excluded if information regarding endoscopy or histology were incomplete or unavailable for analysis.

### Endoscopy and WATS-3D

All patients underwent standard white-light endoscopy (and chromoendoscopy where available) with sampling of the suspected BE segment as per the Seattle protocol and GI societal guidelines^23^ (directed at any focal area of concern and ≥4 samples every 1-2 cm of visible BE including the top of the gastric folds). Patients with endoscopically visible BE were addressed by this standard. If no visible BE was present, FB sampling included 4 samples in at least the distal 1 cm above the neo-squamocolumnar junction and at least 4 samples from the cardia at the top of the gastric folds. The circumferential and longitudinal lengths of any residual BE were reported according to the Prague criteria^35^ at the time of endoscopy. Baseline preablation Prague classification was not available from all records and not specifically addressed.

All patients underwent WATS-3D brush biopsy sampling as per a previously reported standard technique.^28^^,^^29^ As per standard registry protocol, all WATS-3D specimens were sent to CDx Diagnostics central laboratory for processing and analysis by pathologists specifically trained and highly experienced at interpretation of these specimens. One WATS-3D kit was used for every 5 cm of suspected BE. Each kit contains 2 brushes, 1 of which is used to smear cells on a slide subsequently stained with PAP (Papanicolaou stain) and 1 in which the tissue is formalin-fixed and paraffin-embedded into a cellblock and stained with hematoxylin and eosin. The smear is then evaluated with the aid of a high-speed computer and neural network software program specifically optimized for BE tissue that identifies the 200 most suspicious and atypical cellular aggregates. These areas are presented to a GI pathologist on a high-resolution color video monitor after extended depth of field imaging is performed for review at the time of microscopic analysis of the smear and hematoxylin and eosin–stained glass slides.

The extended depth of field imaging was performed as follows. The 150-μm-thick tissue smear was scanned by an extended depth of field imaging system designed to capture 3D tissue fragments. The extended depth of field system captures up to 50 separate optical slices throughout the full thickness of the cell aggregates, at 3-μm intervals, creating multiple 2-dimensional images. These images are stacked sequentially on each other into a single “synthesized” 3D image, which is analyzed by a neural network for selection of the cell aggregates most likely to be dysplastic. The slide was then manually reviewed by a pathologist, addressing special attention to the annotated areas on the synthesized 3D images.

### Pathologic criteria

Standard previously published histologic and cytologic criteria were used to diagnose BE (IM), dysplasia, and EAC in both FB and WATS-3D specimens.^3^^,^^36^ Specimens were classified as NDBE if they contained noncrowded glands lined by columnar cells and goblet cells with retention of the normal honeycomb pattern, cytologically small blind nuclei without hyperchromasia, smooth nuclear contours, a pale/open chromatin pattern, and either small inconspicuous or completely absent nucleoli. Low-grade dysplasia showed loss of surface maturation, loss of the normal honeycomb pattern of cells, increased nuclear-to-cytoplasmic ratio, hyperchromaticity, irregular nuclear contours, increased mitoses, and cell crowding but only mild nuclear stratification and either absent or only mild loss of cell polarity.

Crypt dysplasia was diagnosed according to previously published criteria as well^37^ and was characterized by the presence of low-grade dysplasia epithelium and cell aggregates, as described above, but without evidence of surface involvement by dysplastic epithelium. High-grade dysplasia (HGD) showed more severe nuclear atypia, higher nuclear-to-cytoplasmic ratio and nuclear irregularity, thickened nuclear membranes, atypical mitoses, more pronounced stratification, and more striking loss of cellular polarity. EAC was defined by the presence of tissue invasion.

All pathologists were blinded to the results of the other technique and to the length of the BE segment. All forceps biopsy samples were interpreted locally by GI pathologists. and cases of dysplasia were confirmed by a second expert pathologist as is standard in community practices. All WATS samples were handled centrally with cases of dysplasia confirmed centrally by at least 1 additional expert cytopathologist.

### Statistical analysis

IM adjunctive yield of WATS was defined as the number of IM cases added by WATS (that were negative for IM by FB sampling) divided by the number of IM cases detected by FB sampling (including dysplasia cases). Dysplasia adjunctive yield of WATS was defined as the number of dysplasia cases (indefinite or more advanced) added by WATS (negative by FB sampling for any degree of dysplasia) divided by the number of dysplasia cases detected by FB sampling. The absolute yield of WATS was defined as the number of cases that WATS detected (that were not detected by FB sampling) divided by the total number of cases. The number needed to test was defined as 1/absolute yield.

WATS adjunctive and absolute yield rates were tabulated by segment length into subgroups of length 0 cm, ultrashort (.1-.9 cm), short (1-2.9 cm), and long (≥ 3 cm). Confidence intervals for adjunctive and absolute yield were estimated using Fieller’s theorem^38^ and the binomial approximation to the Gaussian, respectively. *P* values comparing yield rates for different segment length subgroups were calculated using the z-test for proportions^39^ using Microsoft Excel version 2204 (Microsoft, Redmond, Wash, USA) and Wolfram Mathematica version 12.2 (Wolfram Research, Inc, Champaign, Ill, USA). A *P* < .05 was considered statistically significant.

## Results

Table 1 summarizes the clinical and endoscopic characteristics of the patients. A total of 1715 WATS-3D specimens from 1114 patients (mean age, 68.9 years; male-to-female ratio, 68.1%) were analyzed in this study. Most patients were white (88%), had radiofrequency ablation (77.7%), and had a preablation history of NDBE (47.9%), HGD or EAC (18.7%), or indefinite dysplasia, crypt dysplasia, low-grade dysplasia, and/or unspecified dysplasia (33.4%) on prior preablation endoscopies. Most patients also had a hiatus hernia (60.3%). Table 1 also summarizes the length of postablation residual BE noted at the time of the patients first follow-up visit: 88% of patients had evidence of residual BE, of which most were either <1.0 cm (40.1%) or between 1 and 2.9 cm (35.5%) in length.

Table 2 summarizes the adjunctive and absolute yield of WATS-3D for detection of postablation IM (all 1114 patients). Overall, the adjunctive and absolute yield for all endoscopic cases was 52.5% and 16.0%, respectively. The adjunctive yield was statistically higher for patients with short- (or ultrashort) versus long-segment (>3 cm) residual BE. Furthermore, the WATS-3D adjunctive yield was highest among patients in whom there was no endoscopic evidence of residual BE (260%), and this value was significantly greater for patients with endoscopic evidence of BE regardless of length. In contrast, no significant differences were noted in the absolute yield of WATS-3D between patients without versus with visible BE or between patients with short- (or ultrashort) versus long-segment BE.

**Table 2:** Intestinal metaplasia WATS adjunctive and absolute yield

Residual segment length	No. of cases	Adjunctive yield n/N (%; 95% confidence interval)	Absolute yield n/N (95% confidence interval)
All cases	1715	275/524 (52.5; 45.2-60.6)	275/1715 (16.0; 14.3-17.8)
0 cm	205	26/10 (260.0; 132.0-702.8)	26/205 (12.7; 8.1-17.2)
.1-.9 cm (ultrashort)	687	117/175 (66.9; 52.5-84.2)	117/687 (17.0; 14.2-19.8)
1-2.9 cm (short)	608	101/192 (52.6; 40.9-66.5)	101/608 (16.6; 13.7-19.6)
≥3 cm (long)	215	31/147 (21.1; 13.4-29.9)	31/215 (14.4; 9.7-19.1)
*P* value 0 cm vs >0 cm		.001∗	.163
*P* value ultrashort vs short		.162	.841
*P* value short vs long		.001∗	.451

Adjunctive yield = additional cases found by WATS/cases found by forceps biopsy sampling. Absolute yield = additional cases found by WATS/total cases.

*WATS*, Wide-area transepithelial sampling.

∗ Significant *P* < .05.

Table 3 summarizes the adjunctive and absolute yield of detection of dysplasia by WATS-3D. Also noted is the percentage of instances where FB sampling detected dysplasia but WATS-3D detected either a 1- or 2-grade higher level of dysplasia in the same endoscopy. The overall adjunctive yield of dysplasia was 91.5%, but the differences between short- and long-segment BE and between cases either with or without visible BE was not significant. Similar to the results for IM, WATS-3D also detected many cases of dysplasia missed by FB sampling when there was no evidence of residual BE by endoscopy (adjunctive yield of 60.0%). Overall, WATS-3D resulted in an increased grade of dysplasia by either 1 or 2 levels in 9.8% and 3.7% of cases, respectively. The absolute yield of dysplasia was 4.4%. In contrast to adjunctive yield, the absolute yield of dysplasia detection was significantly greater in patients with visible BE versus those without visible BE and between patients with long-segment versus short-segment BE. Additionally, significant differences were found in either adjunctive or absolute yield of WATS-3D for detection of either IM or dysplasia when the data were evaluated according to the number of postablation visits (first vs second vs third etc).

**Table 3:** Dysplasia WATS adjunctive and absolute yield

Residual segment length	No. of cases	Adjunctive yield	Absolute yield	Dysplasia upgrade by WATS	Dysplasia 2-step upgrade by WATS
All cases	1715	75/82 (91.5; 66.3-125.6)	75/1715 (4.4; 3.4-5.3)	8/82 (9.8; 3.0-17.5)	3/82 (3.7; .0-8.2)
0 cm	205	3/5 (60.0; .0-495.8)	3/205 (1.5; .0-3.1)	1/5 (20.0; .0-186.9)	0/5 (.0; .0-.0)
.1-.9 cm (ultrashort)	687	21/21 (100.0; 51.9-192.5)	21/687 (3.1; 1.8-4.3)	1/21 (4.8; .0-16.4)	1/21 (4.8; .0-16.4)
1-2.9 cm (short)	608	19/26 (73.1; 37.3-134.0)	19/608 (3.1; 1.7-4.5)	4/26 (15.4; .4-35.7)	1/26 (3.8;0.0-12.8)
≥3 cm (long)	215	32/30 (106.7; 63.6-180.4)	32/215 (14.9; 10.1-19.6)	2/30 (6.7; .0-17.8)	1/30 (3.3; .0-10.9)
*P* value 0 cm vs >0 cm		.551	.030∗	.488	.659
*P* value ultrashort vs short		.467	.944	.288	.882
*P* value short vs long		.337	.001∗	.346	.921

Values are n/N (%; 95% confidence interval) unless otherwise indicated. Adjunctive yield = additional cases found by WATS/cases found by FB sampling. Absolute yield = additional cases found by WATS/total cases. Dysplasia upgrade = cases where WATS found a worse dysplasia grade than FB sampling / FB sampling dysplasia cases.

*WATS*, Wide-area transepithelial sampling.

∗ Significant *P* < .05.

Table 4 is a contingency matrix summarizing all the precise increases and decreases of pathology grade by category between FB sampling versus WATS. Table 5 outlines the specific diagnoses established by FB sampling and WATS-3D. For instance, in the 29 cases diagnosed as HGD/EAC by WATS-3D, FB sampling diagnosed 18 as HGD/EAC, 2 as crypt dysplasia, 1 as indefinite, 1 as NDBE, and 7 as columnar metaplasia without IM.

**Table 4:** WATS vs FB sampling diagnosis contingency matrix for all cases

	FB sampling diagnosis						
WATS diagnosis	**A**	B	C	D	E	F	Total
**A**. Columnar, no intestinal metaplasia	916	*137*	*3*	*0*	*4*	*1*	1061
B. Intestinal metaplasia	**252**	*253*	*9*	*1*	*4*	*3*	522
C. Indefinite for dysplasia	**2**	*7*	*0*	*0*	*1*	*0*	10
D. Crypt dysplasia	**12**	*34*	*4*	*2*	*7*	*2*	61
E. Low-grade dysplasia	**2**	*10*	*3*	*1*	*15*	*1*	32
F. High-grade dysplasia/adenocarcinoma	**7**	*1*	*1*	*2*	*0*	*18*	29
Total	1191	442	20	6	31	25	1715

Illustration of adjunctive yield calculation for intestinal metaplasia = WATS added cases (in bold)/FB sampling cases (in italic) = 275/524 (= Table 2, row 2, column 3).

*WATS*, Wide-area transepithelial sampling.

**Table 5:** WATS absolute yield before and after further ablation (n = 64)

WATS absolute yield	Visit with ablation on DOS	Next visit	*P* value
Dysplasia	1/64 (1.6; .0-4.6)	1/64 (1.6; .0-4.6)	1.000
Nondysplastic intestinal metaplasia	10/64 (15.6; 6.7-24.5)	11/64 (17.2; 7.9-26.4)	.811

Values are n/N (%; 95% confidence interval). Absolute yield = additional cases found by WATS/total cases.

*WATS*, Wide-area transepithelial sampling; DOS, date of service.

Finally, the data were also analyzed according to the indication for ablation, either NDBE or dysplastic BE. No significant differences were noted in either adjunctive or absolute yield of residual BE between patients with or without preablation dysplasia (Supplementary Tables 1 and 2, available online at www.igiejournal.org) or in the adjunctive or absolute yields of dysplasia (Supplementary Tables 3 and 4, available online at www.igiejournal.org). Note that some patients who did not have dysplasia preablation (and thus were part of the NDBE cohort) did in fact have dysplasia detected postablation (6 detected by FB sampling and an additional 9 detected by WATS-3D).

Table 6 summarizes the number of patients needed to test to detect additional cases of residual BE (6.2 patients) or dysplasia (22.9 patients). A significantly higher number of patients are needed in patients with visible versus no visible BE and between patients with long- versus short-segment BE.

**Table 6:** Wide-area transepithelial sampling number needed to test

Residual segment length	No. of cases	Intestinal metaplasia	Dysplasia
All cases	1715	1715/275 (6.2; 5.6-7.0)	1715/75 (22.9; 18.7-29.4)
0 cm	205	205/26 (7.9; 5.8-12.3)	205/3 (68.3; 32.2-infinity)
.1-.9 cm (ultrashort)	687	687/117 (5.9; 5.0-7.0)	687/21 (32.7; 23.0-56.5)
1-2.9 cm (short)	608	608/101 (6.0; 5.1-7.3)	608/19 (32.0; 22.2-57.4)
≥3 cm (long)	215	215/31 (6.9; 5.2-10.3)	215/32 (6.7; 5.1-9.9)
*P* value 0 cm vs >0 cm		.163	.030∗
*P* value ultrashort vs short		.841	.944
*P* value short vs long		.451	.001∗

Values are n/N (%; 95% confidence interval). Number needed to test = total cases/additional cases found by wide-area transepithelial sampling.

∗ Indicates statistically significant *P* value <.05.

## Discussion

EET has become the treatment of choice for BE patients with dysplasia. However, to prevent recurrences and to detect and eliminate residual disease, continued endoscopic surveillance with biopsy sampling according to the most recent societal guidelines is recommended.^9^^,^^10^ WATS-3D has proven to be effective at increasing the level of detection of BE and dysplasia when added as an adjunct to FB sampling in BE patients undergoing screening and routine surveillance.^28^, ^29^, ^30^, ^31^, ^32^, ^33^ Several studies have addressed post-EET surveillance,^17^, ^18^, ^19^, ^20^, ^21^, ^22^ but none has evaluated WATS-3D other than 1 study that was published only as an abstract.^34^

The purpose of this study was to determine if WATS-3D is effective at increasing the diagnostic yield of BE and dysplasia in the postablation setting. Our results showed that the adjunctive yield of WATS-3D for detection of residual/recurrent BE and dysplasia was overall 52.5% and 91.5%, respectively. The added yield of detection of BE was found to be greater in patients with either no endoscopic evidence of residual BE (260%) or in those with short segments of residual BE (66.9%) compared with long-segment BE (21.1%). However, for dysplasia, no differences were noted according to either the presence or length of visible BE at endoscopy. The absolute yield of BE and dysplasia detection by WATS-3D was 16% and 4.4%, respectively. Although no differences were noted according to the presence or length of residual BE, for dysplasia it was significantly lower in patients with either no BE (1.5%) or only short-segment BE (3.1%) compared with long-segment residual BE (14.9%). The number needed to test for detection of IM and dysplasia was 6.2 and 22.9, respectively, with the addition of WATS-3D to FB sampling. Additionally, there was a 1- or 2-step upgrade in the degree of dysplasia detected by WATS-3D as determined by FB sampling in the same endoscopy in 9.8% and 3.7% of cases, respectively. These data suggest that WATS-3D is a highly effective adjunctive method of surveillance of BE patients after EET and may significantly alter further treatment strategy.

Prior studies that used FB sampling alone have shown that in patients without visible BE, most residual or recurrent IM or dysplasia after EET occurs at the level of the EGJ located at the most proximal aspect of the gastric folds.^18^, ^19^, ^20^ In this study, WATS-3D showed the highest adjunctive yield of IM in precisely this group of patients without visible BE, and the yield was also higher in patients with short- versus long-segment residual BE. Of note, WATS-3D detected either HGD or EAC in 29 patients, whereas FB sampling failed to detect this pathology in 11 of these patients. In addition, FB sampling did not detect IM in 7 of these patients. One possible explanation for these results is that in previous studies of nonablated BE patients, IM has been shown to be less prevalent in the distal versus proximal segments of metaplastic columnar epithelium.^40^^,^^41^ Thus, because WATS-3D samples a broader area of mucosa compared with FB sampling, it is more likely to detect pathologic lesions when the latter are more focal and more widely spatially distributed, such as at the level of the EGJ.

We speculate that WATS-3D may also be particularly helpful in sampling the distal aspect of a stricture or ring and in sampling the cardia, both of which represent areas where endoscopic visualization may be difficult, even with the use of a transparent cap. This remains to be tested. In contrast, the distribution of dysplasia in BE has been shown to be more variable,^11^^,^^42^ and this may explain why, in this study, WATS-3D showed statistically equal, but not higher, adjunctive yield in short- versus long-segment residual BE.

Our results in this large subset of 1114 post-EET patients from the community setting are similar to those reported in an abstract by Iorio et al.^34^ In that study, which included a smaller subset of 110 patients from an academic setting, the incremental yield of WATS-3D was 64.9% for IM and 57.1% for dysplasia when used after EET as an adjunct to FB sampling in patients with no endoscopically visible disease. Further studies are needed to determine whether the added yield of WATS-3D is related in part to an increased ability to sample buried BE in comparison with FB sampling.

A large proportion of the patients in our study had a preablation baseline diagnosis of NDBE (47.6%). We recognize that current GI society guidelines do not endorse EET routinely for patients with NDBE.^9^ However, it is apparent that EET for NDBE is in fact relatively common. This is true not only within community practices but also within academic centers. For instance, in 2015 Iorio et al^34^ reported that 23.6% of their 110 patients had baseline NDBE before EET. Of note is the fact that many patients who had EET for NDBE in our current study had participated in 2 other studies designed specifically to evaluate the efficacy of EET in BE patients either with or without dysplasia (Lyday et al^43^ and Wolf et al^44^). In fact, the study by Wolf et al was specifically designed to evaluate the incidence of cancer, after EET, in patients without dysplasia and in those with various grades of dysplasia. The data showed that EET was significantly more efficacious at preventing cancer development in comparison with the results of prior published studies that evaluated cancer incidence based on routine endoscopic surveillance. Interestingly, we also performed a subanalysis on the patients in this study according to the indication for EET (NDBE vs dysplasia) and found no significant effect on the results. For instance, the differences in the adjunctive yield of IM in the NDBE group was statistically similar to the results obtained in the dysplasia group. Furthermore, 9 patients who had NDBE at baseline (before EET) were found to have dysplasia after EET. In fact, all these dysplasia cases were detected by WATS-3D, but only 6 cases were detected by FB sampling (Supplementary Table 2). These observations would suggest that inclusion of a nondysplastic cohort in this study should not have affected the validity of these results.

Our study had several limitations that need to be highlighted. It is a registry-based study that included multiple centers. Although centers were instructed to sample according to the Seattle protocol, monitoring the use of this protocol was not performed. This analysis was performed on a patient database assembled for commercial use. As such, some variables that might be of clinical relevance (duration of BE, presence of obesity, and use of aspirin, nonsteroidal anti-inflammatory drugs, proton pump inhibitors, etc) were not available. Other variables, such as the presence of a hiatal hernia, BE length at baseline, and number of years post-EET, were incompletely reported. Furthermore, ablation type was also not standardized, although most patients had radiofrequency ablation. In addition, all WATS-3D specimens were interpreted by pathologists at 1 facility (CDx Diagnostics), and the FB sample results were reviewed locally. However, these limitations represent real-world conditions. Some advantages of this study included the prospective nature of the registry studies and the large cohort size.

In summary, our study showed that WATS-3D can be used effectively to increase the diagnostic yield of IM and dysplasia in BE patients after ablation. In fact, the highest yields were achieved in the specific group of patients most difficult to detect clinically and with the known highest frequency of recurrences: those with no residual visible BE. Although EET for BE has been available for more than 2 decades, optimal post-EET surveillance has not yet been defined. Further studies designed to evaluate the utility of WATS-3D, FB sampling, and other techniques for BE detection and surveillance are needed to determine the optimal practice in various post-EET settings.

## Figures and Tables

**Supplementary Table 1:** Intestinal metaplasia WATS adjunctive and absolute yield for cases with preablation indication of nondysplastic Barrett’s esophagus

Residual segment length	No. of cases	Adjunctive yield	Absolute yield
All cases	716	106/168 (63.1; 49.0-80.1)	106/716 (14.8; 12.2-17.4)
0 cm	109	13/1 (1300.0; 342.3-infinite)	13/109 (11.9; 5.8-18.0)
.1-.9 cm (ultrashort)	252	30/66 (45.5; 28.0-68.5)	30/252 (11.9; 7.9-15.9)
1-2.9 cm (short)	293	53/65 (81.5; 55.9-117.3)	53/293 (18.1; 13.7-22.5)
≥3 cm (long)	62	10/36 (27.8; 10.7-51.0)	10/62 (16.1; 7.0-25.3)
*P* value 0 cm vs >0 cm		.001∗	.358
*P* value ultrashort vs short		.041∗	.045∗
*P* value short vs long		.006∗	.714

Values are n/N (%; 95% confidence interval). Adjunctive yield = additional cases found by WATS/cases found by FB sampling. Absolute yield = additional cases found by WATS/total cases.

*WATS*, Wide-area transepithelial sampling.

∗ Significant *P* < .05.

**Supplementary Table 2:** Adjunctive and absolute yield of WATS-3D for detection of dysplasia in patients without dysplasia preablation

Residual segment length	No. of cases	Adjunctive yield	Absolute yield	Dysplasia upgrade by WATS	Dysplasia 2-step upgrade by WATS
All cases	716	9/6 (150.0; 45.7-780.4)	9/716 (1.3; .4-2.1)	0/6 (.0; .0-.0)	0/6 (.0; .0-.0)
0 cm	109	0/0 (—)	0/109 (.0; .0-.0)	0/0 (—)	0/0 (—)
.1-.9 cm (ultrashort)	252	3/2 (150.0; .0-315.4)	3/252 (1.2; .0-2.5)	0/2 (.0; .0-.0)	0/2 (.0; .0-.0)
1-2.9 cm (short)	293	3/3 (100.0; .0-744.3)	3/293 (1.0; .0-2.2)	0/3 (.0; .0-.0)	0/3 (.0; .0-.0)
≥3 cm (long)	62	3/1 (300.0; .0-192.5)	3/62 (4.8; .0-10.2)	0/1 (.0; .0-.0)	0/1 (.0; .0-.0)
*P* value 0 cm vs >0 cm		—	.201	—	—
*P* value ultrashort vs short		.740	.853	—	—
*P* value short vs long		.429	.034∗	—	—

Values are n/N (%; 95% confidence interval). Adjunctive yield = additional cases found by WATS/cases found by FB sampling. Absolute yield = additional cases found by WATS/total cases. Dysplasia upgrade = cases where WATS finds a worse dysplasia grade than FB sampling/FB sampling dysplasia cases.

*WATS*, Wide-area transepithelial sampling.

∗ Significant *P* < .05.

**Supplementary Table 3:** Intestinal metaplasia WATS adjunctive and absolute yield for cases with preablation indication of dysplasia

Residual segment length	No. of cases	Adjunctive yield	Absolute yield
All cases	999	169/356 (47.5; 39.3-56.7)	169/999 (16.9; 14.6-19.2)
0 cm	96	13/9 (144.4; 58.7-431.2)	13/96 (13.5; 6.7-20.4)
.1-.9 cm (ultrashort)	435	87/109 (79.8; 59.7-105.7)	87/435 (20.0; 16.2-23.8)
1-2.9 cm (short)	315	48/127 (37.8; 26.2-51.7)	48/315 (15.2; 11.3-19.2)
≥3 cm (long)	153	21/111 (18.9; 10.7-28.5)	21/153 (13.7; 8.3-19.2)
*P* value 0 cm vs >0 cm		.006∗	.354
*P* value ultrashort vs short		.001∗	.094
*P* value short vs long		.017∗	.665

Values are n/N (%; 95% confidence interval). Adjunctive yield = additional cases found by WATS/cases found by FB sampling. Absolute yield = additional cases found by WATS/total cases.

*WATS*, Wide-angle transepithelial sampling.

∗ Significant *P* < .05.

**Supplementary Table 4:** Dysplasia WATS adjunctive and absolute yield for cases with preablation indication of dysplasia

Residual segment length	No. of cases	Adjunctive yield	Absolute yield	Dysplasia upgrade by WATS	Dysplasia 2-step upgrade by WATS
All cases	999	66/76 (86.8; 61.8-121.1)	66/999 (6.6; 5.1-8.1)	8/76 (10.5; 3.2-19.0)	3/76 (3.9; .0-8.8)
0 cm	96	3/5 (60.0; .0-466.2)	3/96 (3.1; .0-6.6)	1/5 (20.0; .0-176.5)	0/5 (.0; .0-.0)
.1-.9 cm (ultrashort)	435	18/19 (94.7; 46.5-190.5)	18/435 (4.1; 2.3-6.0)	1/19 (5.3; .0-18.4)	1/19 (5.3; .0-18.4)
1-2.9 cm (short)	315	16/23 (69.6; 33.1-133.5)	16/315 (5.1; 2.7-7.5)	4/23 (17.4; .4-41.2)	1/23 (4.3; .0-14.7)
≥3 cm (long)	153	29/29 (100.0; 58.4-171.3)	29/153 (19.0; 12.7-25.2)	2/29 (6.9; .0-18.4)	1/29 (3.4; .0-11.3)
*P* value 0 cm vs >0 cm		.600	.149	.536	.646
*P* value ultrashort vs short		.504	.541	.281	.895
*P* value short vs long		.385	.001∗	.297	.872

Values are n/N (%; 95% confidence interval). Adjunctive yield = additional cases found by WATS/cases found by FB sampling. Absolute yield = additional cases found by WATS/total cases. Dysplasia upgrade = cases where WATS finds a worse dysplasia grade than FB sampling / FB sampling dysplasia cases.

*WATS*, Wide-area transepithelial sampling.

∗ Significant *P* < .05.
